# Unaltered intrinsic functional brain architecture in young women with primary dysmenorrhea

**DOI:** 10.1038/s41598-018-30827-6

**Published:** 2018-08-28

**Authors:** Lin-Chien Lee, Yueh-Hua Chen, Chia-Shu Lin, Wei-Chi Li, Intan Low, Cheng-Hao Tu, Chih-Che Chou, Chou-Ming Cheng, Tzu-Chen Yeh, Li-Fen Chen, Hsiang-Tai Chao, Jen-Chuen Hsieh

**Affiliations:** 1 Institute of Brain Science, School of Medicine, National Yang-Ming University, Taipei, Taiwan; 2 Integrated Brain Research Unit, Division of Clinical Research, Department of Medical Research, Taipei Veterans General Hospital, Taipei, Taiwan; 3 Department of Physical Medicine and Rehabilitation, Cheng Hsin General Hospital, Taipei, Taiwan; 4 Department of Dentistry, School of Dentistry, National Yang-Ming University, Taipei, Taiwan; 5 Institute of Biomedical Informatics, School of Medicine, National Yang-Ming University, Taipei, Taiwan; 6 Graduate Institute of Acupuncture Science, College of Chinese Medicine, China Medical University, Taichung, Taiwan; 7 Department of Radiology, Taipei Veterans General Hospital, Taipei, Taiwan; 8 Department of Obstetrics and Gynecology, School of Medicine, National Yang-Ming University, Taipei, Taiwan; 9 Department of Obstetrics and Gynecology, Taipei Veterans General Hospital, Taipei, Taiwan

## Abstract

Primary dysmenorrhea (PDM), painful menstruation without organic causes, is the most prevalent gynecological problem in women of reproductive age. Dysmenorrhea later in life often co-occurs with many chronic functional pain disorders, and chronic functional pain disorders exhibit altered large-scale connectedness between distributed brain regions. It is unknown whether the young PDM females exhibit alterations in the global and local connectivity properties of brain functional networks. Fifty-seven otherwise healthy young PDM females and 62 age- and education-matched control females participated in the present resting-state functional magnetic resonance imaging study. We used graph theoretical network analysis to investigate the global and regional network metrics and modular structure of the resting-state brain functional networks in young PDM females. The functional network was constructed by the interregional functional connectivity among parcellated brain regions. The global and regional network metrics and modular structure of the resting-state brain functional networks were not altered in young PDM females at our detection threshold (medium to large effect size differences [Cohen’s *d* ≥ 0.52]). It is plausible that the absence of significant changes in the intrinsic functional brain architecture allows young PDM females to maintain normal psychosocial outcomes during the pain-free follicular phase.

## Introduction

Primary dysmenorrhea (PDM), painful menstruation without organic causes, affects more than one-half of menstruating women worldwide^1^. PDM has been regarded as a genuine type of chronic pelvic pain^1^ (please also refer to the World Health Organization [WHO] website, The 11^th^ Revision of the International Classification of Diseases [ICD-11]; http://www.who.int/classifications/icd/revision/en/). Dysmenorrhea is associated with decreased self-rated overall health^2^ in combination with depressive and anxious symptoms^3^. Otherwise healthy young females with PDM can exhibit a much higher prevalence of incidental brain findings, particularly normal variants (e.g., cavum septum pellucidum), than females without PDM^4^. Furthermore, we previously reported that long-term PDM is associated with alterations in regional metabolism of the brain^5^ and both *state-* and *trait-related* changes in brain structures^6,7^. *State-related* changes are menstrual pain-primed, whereas *trait-related* changes exist even without menstrual pain. We have also reported that the *BDNF* Val66Met polymorphism, may not only contribute to the susceptibility of females to PDM^8^, but also affect the functional connectivity dynamics of the brain in young PDM females^9^.

Recent neuroimaging studies of PDM have disclosed the functional connectivity of the resting-state brain networks undergoes maladaptive or adaptive reorganizations in response to long-term dysmenorrhea. PDM females can exhibit aberrant functional connectivity between regions within the default mode network (DMN)^10^. Previous studies also showed long-term PDM is associated with hypo-connectivity of DMN-descending pain modulatory systems (DPMS)^11^ and -salience network^12,13^ and hyper-connectivity of DMN-executive control network^12^. Collectively, these reorganizations of functional connectivity, both within and across the resting-state brain networks, may underpin the pathophysiological mechanisms of PDM as well as the neural bases of associated sensory and affective elements^11–13^.

The antecedent maladaptive hypo-connectivity of the DPMS with the DMN in PDM may predispose vulnerable PDM females to subsequent development of chronic functional pain disorders^11^. Dysmenorrhea later in life often co-occurs with many chronic functional pain disorders, including fibromyalgia, irritable bowel syndrome, painful bladder syndrome, chronic headache, and chronic low back pain^1^. The highest prevalence rates of chronic functional pain disorders usually occur after age of 30^14^, and the prevalence of PDM peaks much younger in age^15^. In terms of the complex and multidimensional nature of chronic pain and its comorbidities (depression, sleep disorder, and cognitive dysfunction^16^), the maladaptive neuroplasticity of the brain in chronic pain disorders is not confined to the changes in certain brain regions and systems, but can also be manifested in the topological organization of whole-brain networking^17^. Chronic pain disorders, such as fibromyalgia^18^, irritable bowel syndrome^19^, chronic back pain^20^, and migraine^21–23^, exhibit altered large-scale connectedness between distributed brain regions. It has been shown that the topological metrics of whole-brain networking can be correlated with the clinical characteristics of chronic pain disorders (e.g., disease duration)^21^.

The human brain is considered one integrative complex network or system, comprising multiple sub-networks^24^. The global network organization of the brain can be viewed as a dynamic neural correlate of the overall pain experience consisted of nociceptive, cognitive, and affective dimensions^25^. Graph theoretical network analysis provides a mathematical framework to examine the topology of complex networks^24^. It has been used to explore and quantify the global and local organizations of brain networks and to evaluate the integrity of the brain in altered cognitive states (e.g., tasks of different levels of cognitive demand), altered conditions (e.g., aging and sleep), and pathological diseases (e.g., neuropsychiatric diseases)^24,26,27^. In graph theory, the brain networks are composed of nodes (brain regions) and edges (connections between brain regions)^26^. The human brain is organized in a small-world modular structure that confers efficient processing of parallel information^28^. Small-world organization features a high level of local connectedness and a short average travel distance between nodes, with network communities (sub-networks or modules) interlinked by hub regions. The hub is pivotal for interregional communication and integration, and the node degree, a measure of the number of links connected to that node^29^, is used to index the importance of a node as a functional hub in the network^30^. The modular structure reveals how the related brain regions coordinate activity among each other in network communities^29^. The integrity of the brain architecture is indicated by the degree of small-worldness and network efficiency^24^.

Although studies reported alterations in functional connectivity dynamics of the DPMS and DMN in young PDM females^10–13^, it is unknown whether such alterations would influence the intrinsic functional brain architecture. In this study, we used graph theory and resting-state functional magnetic resonance imaging (fMRI) to investigate the influences of long-term PDM on the global and regional network metrics and modular structure of brain functional networks in otherwise healthy young PDM females.

## Results

### Demographic data and psychological assessments

There were no significant between-group differences regarding age (PDM: 23.1 ± 2.27 years of age, control: 23.7 ± 2.40 years of age, *P* = 0.147), age at menarche (PDM: 12.2 ± 1.19 years of age, control: 12.2 ± 1.11 years of age, *P* = 0.811), years of menstruating (PDM: 10.9 ± 2.53 years, control: 11.5 ± 2.69 years, *P* = 0.194), or average duration of one menstrual cycle (PDM: 29.3 ± 1.41 days, control: 29.5 ± 1.19 days, *P* = 0.525). The PDM group had a long history of menstrual pain (8.8 ± 2.75 years), with the pain lasting approximately 1 to 3 days during one menstrual cycle (2.0 ± 0.84 days). The current menstrual pain experience, as assessed by the pain rating index (29.3 ± 12.70) and present pain intensity (2.6 ± 1.01) of McGill Pain Questionnaire, confirmed that the PDM group experienced moderate to severe menstrual pain. The PDM females reported significantly higher *state* anxiety, *trait* anxiety, Beck Anxiety Inventory, and Pain Catastrophizing Scale scores during both the menstrual phase (MENS phase) and periovulatory phase (POV phase) (Table 1).

**Table 1:** Demographic data and baseline information of the PDM and CON groups.

	PDM (n = 57)	CON (n = 62)	*P* value
Age, year	23.1 ± 2.27	23.7 ± 2.40	0.147
Age at menarche	12.2 ± 1.19	12.2 ± 1.11	0.811
Years of menstruating	10.9 ± 2.53	11.5 ± 2.69	0.194
Days of one menstrual cycle	29.3 ± 1.41	29.5 ± 1.19	0.525
Menstrual pain experience			
Years of dysmenorrhea history	8.8 ± 2.75		
Days of menstrual pain per cycle	2.0 ± 0.84		
Overall PRI (inception of study; range, 0–78)	34.9 ± 13.35		
Overall PPI (inception of study; range, 0–5)	3.1 ± 1.11		
Current PRI (MENS phase; range, 0–78)*	29.3 ± 12.70		
Current PPI (MENS phase; range, 0–5)*	2.6 ± 1.01		
Beck Anxiety Inventory (range, 0–63)			
MENS phase	12.0 ± 7.87	2.4 ± 2.38	<0.001
POV phase	6.4 ± 5.50	3.2 ± 3.37	<0.001
State-Trait Anxiety Inventory: *State* (range, 20–80)			
MENS phase	43.1 ± 9.03	34.1 ± 7.35	<0.001
POV phase	36.5 ± 7.10	34.1 ± 7.68	0.036
State-Trait Anxiety Inventory: *Trait* (range, 20–80)			
MENS phase	44.9 ± 8.87	37.8 ± 7.23	<0.001
POV phase	43.0 ± 8.40	37.8 ± 7.61	0.001
Beck Depression Inventory (range, 0–63)			
MENS phase	11.5 ± 7.92	4.5 ± 4.89	<0.001
POV phase	6.1 ± 6.31	4.3 ± 5.53	0.070
Pain Catastrophizing Scale (range, 0–52)			
MENS phase	21.3 ± 11.60	5.4 ± 5.89	<0.001
POV phase	18.6 ± 10.78	5.9 ± 7.23	<0.001
Estradiol (pg/mL)			
MENS phase	35.4 ± 18.52	43.3 ± 30.13	0.322
POV phase	157.9 ± 111.21	144.7 ± 121.66	0.315
Progesterone (ng/mL)			
MENS phase	0.5 ± 0.39	1.0 ± 2.41	0.368
POV phase	0.9 ± 1.19	1.3 ± 2.83	0.258
Testosterone (ng/mL)			
MENS phase	0.4 ± 0.23	0.4 ± 0.23	0.597
POV phase	0.6 ± 0.31	0.5 ± 0.22	0.369

PDM, primary dysmenorrhea; CON, control; PPI, present pain intensity of the McGill Pain Questionnaire; PRI, pain rating index of the McGill Pain Questionnaire; MENS phase, menstrual phase; POV phase, periovulatory phase. The data are presented as the means ± SD.

*Five PDM subjects did not complete the McGill Pain Questionnaire during the MENS phase and were excluded from the calculation.

### Serum gonadal hormone measurements

No significant between-group differences were found in the concentrations of estradiol, progesterone, and testosterone during either the MENS phase or POV phase (Table 1).

### Global network metrics of the resting-state functional network

**Figure 1:**
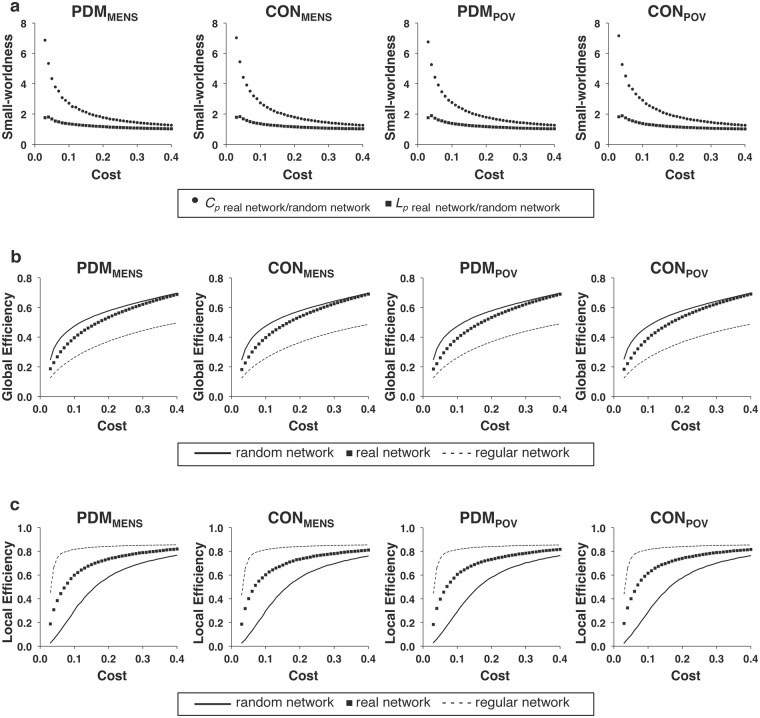
The global network metrics of the weighted network. The global network metrics of (**a**) small-worldness (*C*_*p*_ and *L*_*p*_), (**b**) global efficiency, and (**c**) local efficiency in the weighted network are plotted over the range of network costs (0.03–0.40). No significant differences were found in the global network metrics among the PDM_MENS_, CON_MENS_, PDM_POV_, and CON_POV_ by conducting linear mixed models. *C*_*p*_, clustering coefficient; *L*_*p*_, characteristic path length; CON_MENS_, menstrual phase of the control group; CON_POV_, periovulatory phase of the control group; PDM_MENS_, menstrual phase of the primary dysmenorrhea group; PDM_POV_, periovulatory phase of the primary dysmenorrhea group.

**Figure 2:**
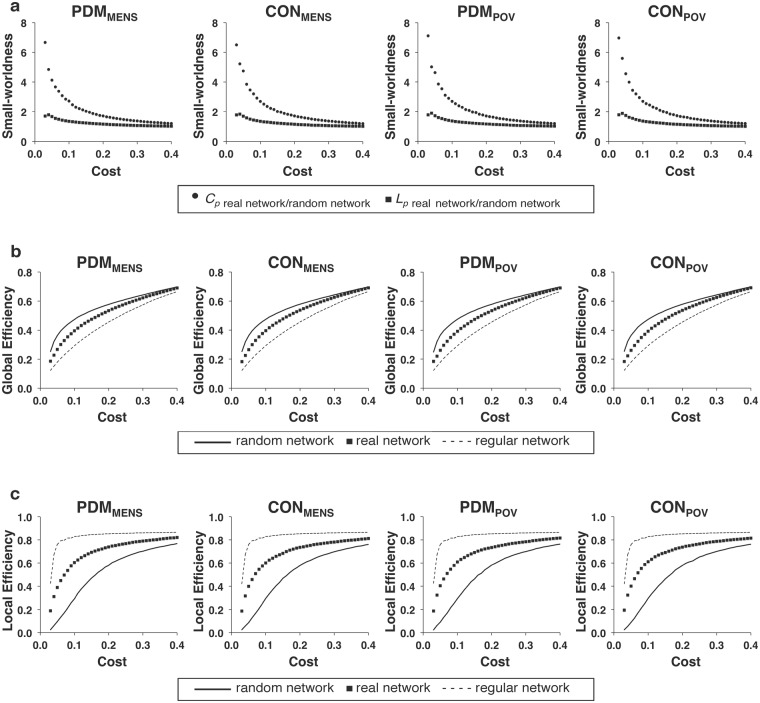
The global network metrics of the binary network. For the full range of network costs (0.03–0.40) of the binary network, no significant differences were found in the global network metrics among the PDM_MENS_, CON_MENS_, PDM_POV_, and CON_POV_ by conducting linear mixed models. *C*_*p*_, clustering coefficient; *L*_*p*_, characteristic path length; CON_MENS_, menstrual phase of the control group; CON_POV_, periovulatory phase of the control group; PDM_MENS_, menstrual phase of the primary dysmenorrhea group; PDM_POV_, periovulatory phase of the primary dysmenorrhea group.

Since the stringent statistical correction for multiple comparisons revealed negative findings, we therefore adopted a liberal uncorrected approach for the following analyses in order to unravel possible subtle effects. For the respective weighted and binary networks (covariates of gonadal hormones and psychological status adjusted), no main effect of group, menstrual cycle phase, or interaction between them was noted for any of the global network metrics (mean clustering coefficient, characteristic path length, global efficiency, and local efficiency of the network) with cost values ranging from 0.03 to 0.40 (all *P* > 0.05, uncorrected for multiple comparisons of 38 cost levels) (Fig. 1: weighted network; Fig. 2: binary network). For the definitions of mean clustering coefficient, characteristic path length, global efficiency, and local efficiency of the network, please refer to the Methods section (see Global network metrics of the resting-state functional network). Small-worldness of brain functional network was confirmed for each phase of both the PDM and control groups. The general efficiency and small-worldness property of the resting-state brain functional network were not altered by acute menstrual pain in young PDM females.

### Modular structure

**Figure 3:**
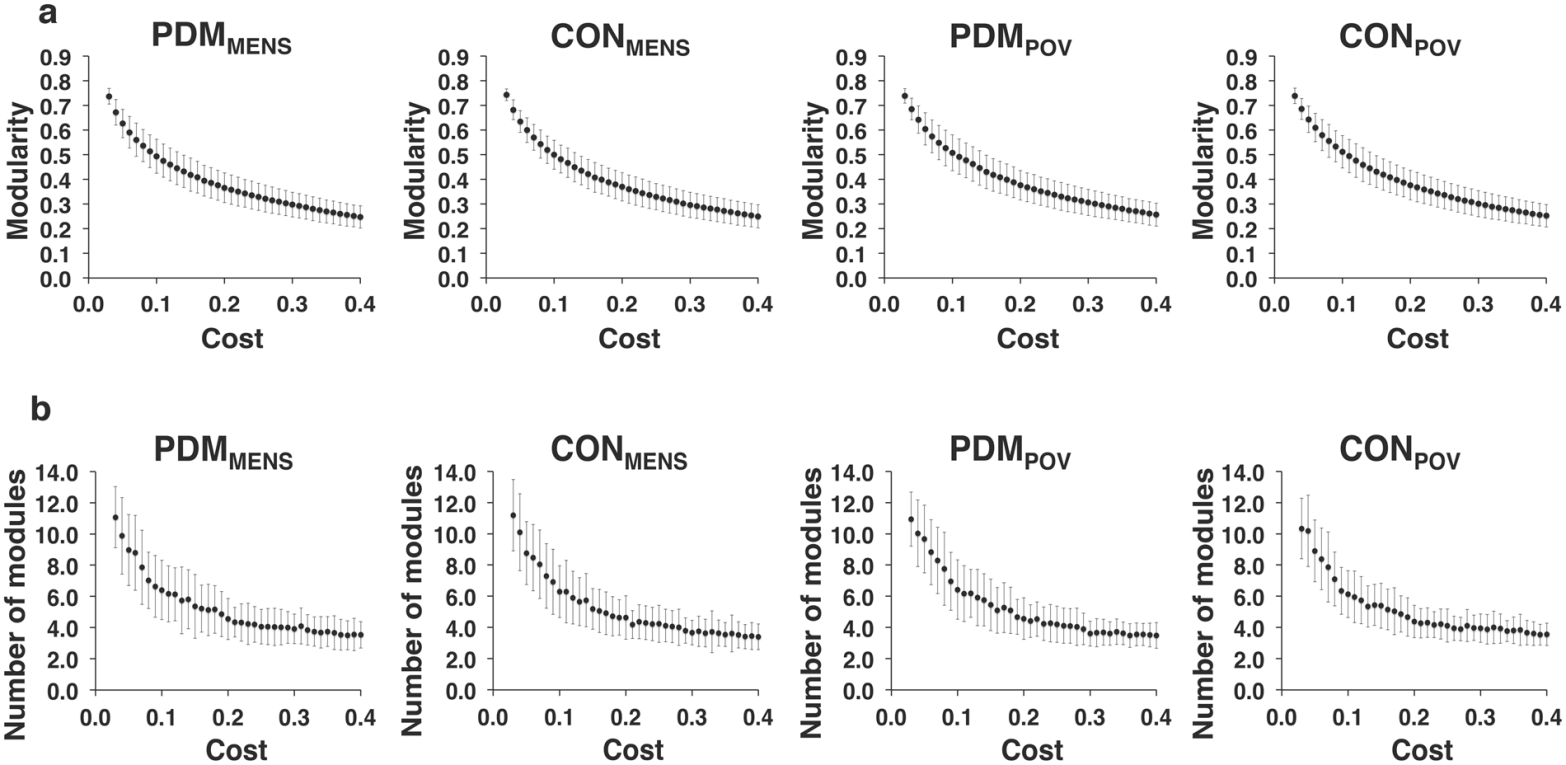
The modular structure of the weighted network. For the full range of network costs (0.03–0.40) of the weighted network, no significant differences were found in (**a**) the modularity and (**b**) the number of partitioned modules among the PDM_MENS_, CON_MENS_, PDM_POV_, and CON_POV_ by conducting linear mixed models. The bar denotes the standard deviation of means. CON_MENS_, menstrual phase of the control group; CON_POV_, periovulatory phase of the control group; PDM_MENS_, menstrual phase of the primary dysmenorrhea group; PDM_POV_, periovulatory phase of the primary dysmenorrhea group.

**Figure 4:**
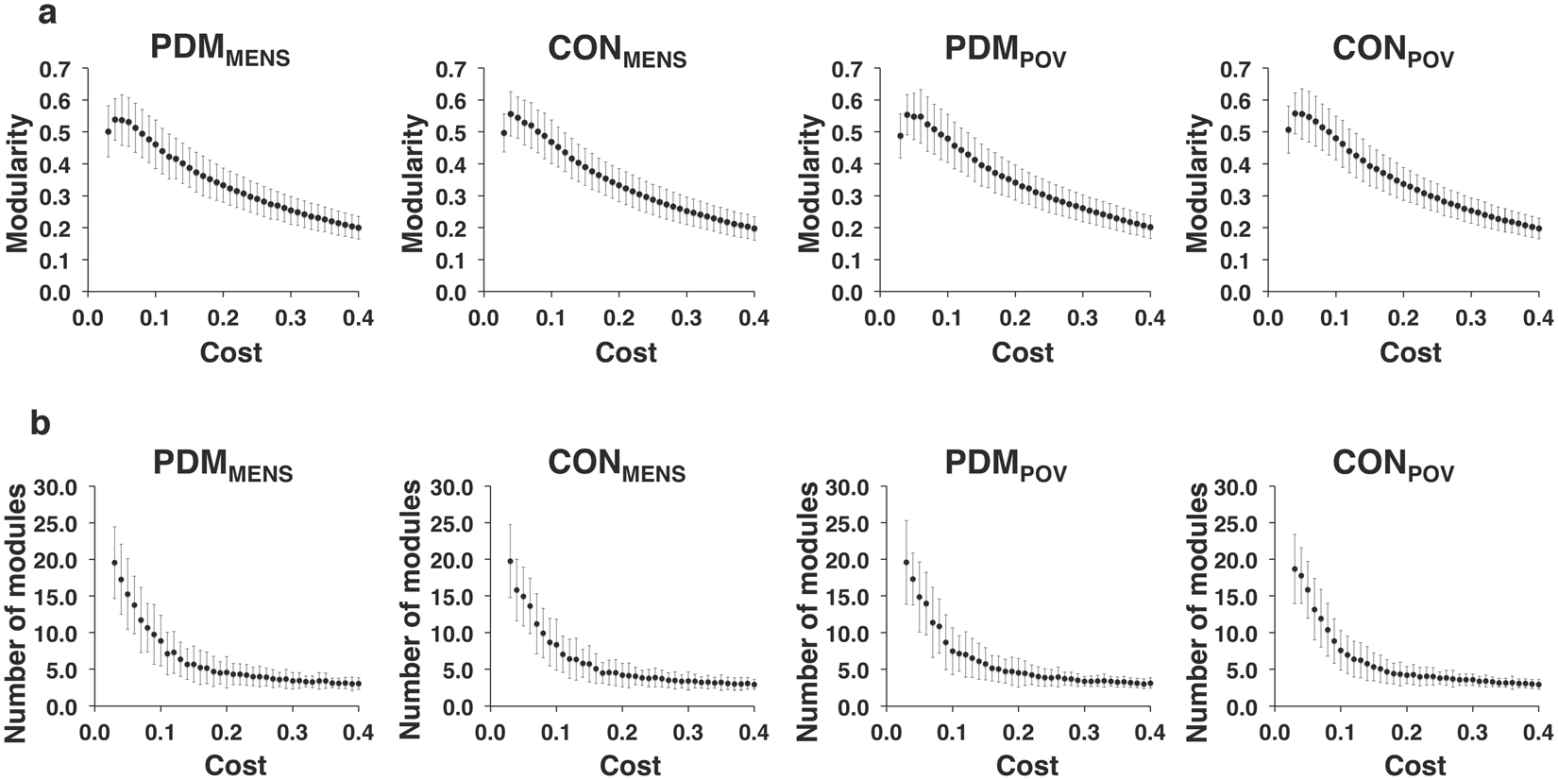
The modular structure of the binary network. For the full range of network costs (0.03–0.40) of the binary network, no significant differences were found in (**a**) the modularity and (**b**) the number of partitioned modules among the PDM_MENS_, CON_MENS_, PDM_POV_, and CON_POV_ by conducting linear mixed models. The bar denotes the standard deviation of means. CON_MENS_, menstrual phase of the control group; CON_POV_, periovulatory phase of the control group; PDM_MENS_, menstrual phase of the primary dysmenorrhea group; PDM_POV_, periovulatory phase of the primary dysmenorrhea group.

**Figure 5:**
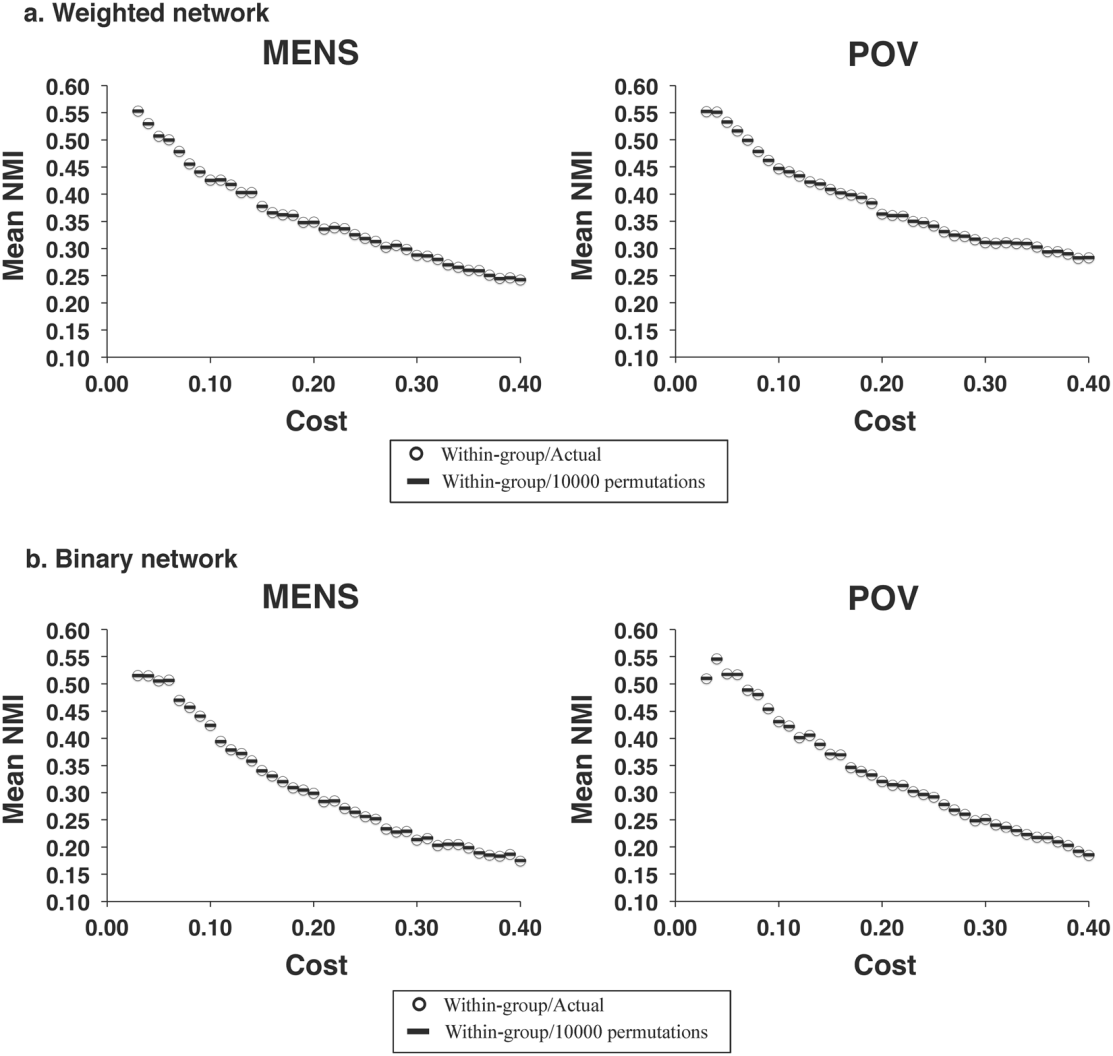
The similarity of modular partitions during the same menstrual cycle phases in the respective weighted and binary networks. For the full range of network costs (0.03–0.40) of the respective (**a**) weighted and (**b**) binary networks, no significant differences were found in the similarity of modular structure for the between-group comparisons during each of the MENS and POV phases. MENS, menstrual phase; NMI, normalized mutual information; POV, periovulatory phase.

**Figure 6:**
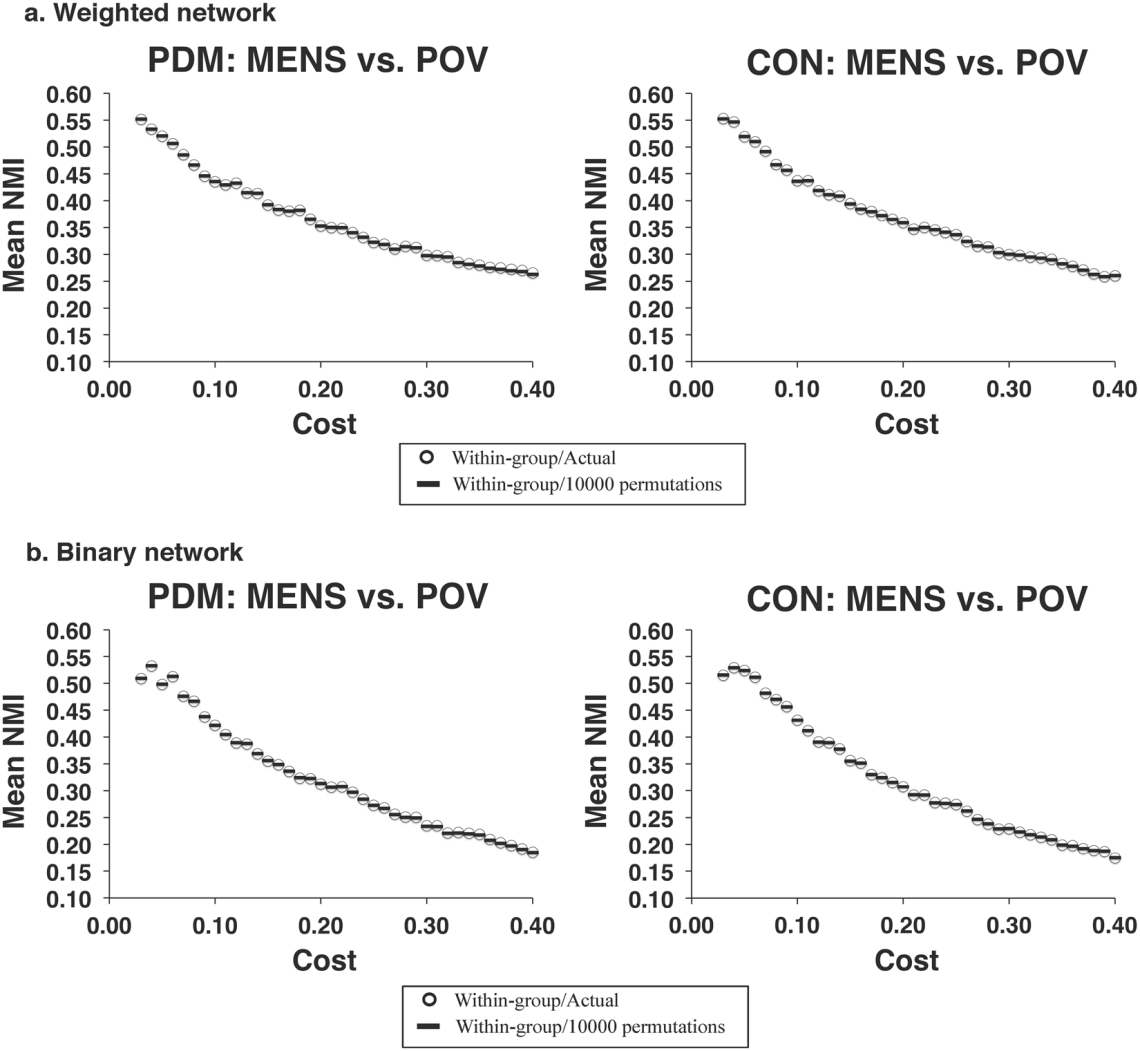
The similarity of modular partitions for the between-phase comparisons in the respective PDM and CON groups. For the full range of network costs (0.03–0.40) of the respective (**a**) weighted and (**b**) binary networks, no significant differences were found in the similarity of modular structure for the between-phase comparisons in the PDM and CON groups. CON, control; MENS, menstrual phase; NMI, normalized mutual information; PDM, primary dysmenorrhea; POV, periovulatory phase.

For the full range of network costs (0.03–0.40) (covariates of gonadal hormones and psychological status adjusted), no main effect of group, menstrual cycle phase, or interaction between them was noted for the modularity and the number of partitioned modules in the respective weighted and binary networks (all *P* > 0.05, uncorrected for multiple comparisons of 38 cost levels) (Fig. 3: weighted network; Fig. 4: binary network). By assessing normalized mutual information (NMI) at a specific cost level, no significant differences were found in the similarity of modular partitions for the between-group comparisons during each of the MENS and POV phases (Fig. 5) (all *P* > 0.05, uncorrected for multiple comparisons of 38 cost levels). Moreover, no significant differences were found in the similarity of modular partitions for the between-phase comparisons in the respective PDM and control groups (Fig. 6) (all *P* > 0.05, uncorrected for multiple comparisons of 38 cost levels). For the definitions of modularity and NMI, please refer to the Methods section (see Modular structure). The modular structures of the PDM and control groups were found to be similar within the same respective phase.

### Modular assignment of specific nodes

For the full range of network costs (0.03–0.40) in the respective weighted and binary networks, no significant differences were found in the modular assignment of specific nodes for the between-group comparisons during each of the MENS and POV phases (all *P* > 0.05 for 90 nodes, adjusted for False Discovery Rate [FDR] correction of 90 nodes but uncorrected for multiple comparisons of 38 cost levels). Within the same respective phase, the network communities of specific nodes (90 cerebral regions of the Automated Anatomical Labeling [AAL] atlas^31^) were not substantially altered in the PDM group as compared with the control group.

### Regional network metrics of the resting-state functional network

For the range of network costs (0.03–0.10) (covariates of gonadal hormones and psychological status adjusted), no main effect of group, menstrual cycle phase, or interaction between them was noted for the local efficiency, clustering coefficient, and degree of each node in the respective weighted and binary networks (all *P* > 0.05 for 90 nodes, adjusted for FDR correction of 90 nodes but uncorrected for multiple comparisons of 8 cost levels) [Supplementary Table S1: weighted network; Supplementary Table S2: binary network]. For the definition of node degree, please refer to the Methods section (see Regional network metrics of the resting-state functional network). The local connectivity property of brain functional networks in terms of node-level connectivity analyses was not altered by acute menstrual pain in young PDM females.

### Robustness of methodological variation

The findings derived from the Harvard-Oxford cortical and subcortical probabilistic atlases (from FSLView v3.1; http://fsl.fmrib.ox.ac.uk/fsl/fslview) were consistent with those derived from the AAL atlas [Supplementary Figs S1–S6; Supplementary Tables S3 and S4].

## Discussion

In this study, we examined the global and regional network metrics and modular structure of brain functional networks in young PDM females using networks constructed by parcellated cerebral regions according to the AAL and Harvard-Oxford cortical and subcortical probabilistic atlases. No significant between-group differences were found for the metrics of global and local network efficiency of information transfer between nodes. It indicates that the young PDM females may retain the integrity of global and local connectivity properties of brain functional networks despite the presence of maladaptive neuroplasticity of DPMS^11^ and DMN^10,12,13^. Although the young PDM females in our study exhibited higher levels of depressive mood and anxiety compared to the controls (Table 1), these levels did not reach the degree of clinical severity, such as an anxiety disorder that is mandatory for psychiatric intervention. The average Beck Depression Inventory score during the MENS phase in the PDM group was conventionally interpreted as minimal depression^32^, and the average Beck Anxiety Inventory score as mild anxiety^33^.

In terms of unaltered global and regional network metrics and modular structure of brain functional networks in young PDM females, the negative findings support that PDM is essentially dissimilar to major neurological^34^ and psychiatric diseases^35–38^. This is particularly important because otherwise healthy PDM females usually do not exhibit overt cognitive, affective and psychosocial liability and disability, despite the fact that the PDM females may exhibit minimal or mild depressive and anxious symptoms during the MENS phase^3,8^. In this study, we deliberately excluded the females with severe premenstrual syndrome or suspected premenstrual dysphoric disorder, which features a combination of cyclical cognitive and affective debilitations and physical symptoms and is associated with significant functional impairments for daily living^39,40^. Premenstrual dysphoric disorder has been regarded as a psychiatric disease^40^, and psychiatric diseases may exhibit disrupted topological organization of large-scale structural and functional brain networks^36,41^. The absence of overt psychological and psychiatric disturbance is succinctly reflected by the unaltered global and local connectivity properties of brain functional networks in young PDM females. It is documented that the small-world architecture and optimal topological organization of human brain networks are prominently altered in depression^36,37^, social anxiety disorder^38^, Alzheimer’s disease^34,42–44^, and schizophrenia^35,41,45,46^, and the graph metrics can be correlated with the symptom severity of diseases^34,37,42^. These neurological and psychiatric diseases not only target distributed brain regions but also disrupt the topological configuration of large-scale brain networks^24,26^.

Although previous studies reported alterations in local connectivity of the brain in young PDM females by means of regional homogeneity^12,47^ and seed-based functional connectivity analyses^10–13^, the current study showed unaltered local connectivity property in the context of regional network metrics by using graph theoretical network analysis. The discrepancies may be inherent in the different methodologies used. Graph theoretical network analysis quantifies the functional connectivity profiles among node neighborhoods in the topological space and provides the information of connection pattern on a whole-brain scale^24^. Seed-based approach compares the region-to-region functional connectivity in the network, and can only provide the information that is limited to the connection pattern of the selected seed region^24^. Regional homogeneity analysis measures the extent of functional synchronizations among the neighboring voxels and provides the information of connection pattern on a local spatial scale^48^.

The abnormal functional connectivity and networking of the brain in chronic pain disorders cast light on the neurophysiological mechanisms underlying pain chronification and the different facets of associated neurocognitive conditions^18,49–52^. We speculate that the alterations in functional connectivity in young and otherwise healthy PDM females can be initially limited to certain systems of the brain (e.g., maladaptive functionality of the DPMS^11^ and the concomitant adaptive reorganization of cross-network connectivities^12^), without the disruption of topology of brain functional networks. Dysmenorrhea can be associated with high rates of comorbidity with chronic functional pain disorders later in life^1^, and chronic functional pain disorders prominently exhibit not only hypo-connectivity of the DPMS with the DMN^18,51,53^ but also alteration of topology in the whole-brain networking^18–22^. In addition, the extent of alterations of functional connectivity in migraine, a disease that is often comorbid with dysmenorrhea, proceeds from local systems to topological organization of whole-brain networking as the disease duration increased^22^. Chronification of pain involves large-scale, dynamic reorganization of brain connectivities since the transition from acute to chronic pain parallels the transition of brain activation from nociceptive to emotional and reward circuits^54^. We have also recently reported that genetic factors, such as the *BDNF* Val66Met^9^ and *OPRM1* A118G^55^ polymorphisms, may affect, in the genotype-specific process, the functional connectivity dynamics of the DPMS in young PDM females. Such brain resilience that vary with the genotypes contributes to individual pain differences and susceptibility, which in turn may have an influence on the vulnerability for the subsequent development of chronic pain disorders. It is tempting to propose that the cumulative aberrancy of the dysfunctional DPMS^11^ and the repetitive overstimulation of menstrual pain may eventually lead to the alteration of topology of brain architecture in later life of vulnerable PDM females through the chronification processes and genetic attributions. Longitudinal follow-up is needed to investigate the possibly progressive alterations in functional brain architecture of PDM females, and therefore to improve the understanding of the co-occurrence of chronic functional pain disorders.

The correlations between the regional structural changes in gray matter volume and topology of resting-state brain functional networks in PDM females are of interest for further investigation. One may speculate that the patterns of regional structural and functional alterations in the brain shown in previous PDM studies^5–7,10–13,56^ represent a disordered configuration and will translate into abnormal interregional functional connectivity in terms of large-scale networking. The cingulate cortex, a brain region integrating pain, negative emotions and cognition^57^, exhibits *trait-related* changes in glucose metabolism and *state-* and *trait-related* changes in gray matter volume in PDM females^5–7^. Medial brain motor areas, including the supplementary motor area, exhibit altered functional connectivity in chronic pain conditions that are often comorbid with dysmenorrhea, including painful bladder syndrome^58^ and localized provoked vulvodynia^59^. However, the modular assignments and regional network metrics of anterior and posterior cingulate gyri and supplementary motor area parsed based on the AAL and Harvard-Oxford cortical and subcortical probabilistic atlases were not altered in this study. The interactions between functional networks (functional connectivity) and their structural neural substrates of PDM remain to be explored, although functional and structural brain networks are closely related and may share common topological characteristics^24,60,61^. Different brain imaging modalities assess distinct but complementary types of brain connectivity. Integrated models of structural and functional connectivity constructed from multimodal brain imaging data can further shed light on the effect of PDM on topological network architecture.

By using power analysis (G*Power software; http://www.gpower.hhu.de/), the sample size of our study (n = 119 in total and ~60 in each group) may achieve statistical power (80%) to detect the medium effect size larger than 0.52 for the between-group/within-phase comparison (independent *t* test) and 0.37 for the within-group/between-phase comparison (paired *t* test). However, statistical significance in our study cannot be achieved should the effect size is smaller (e.g., underpowered concern in our statistical construct). Possible existence of more subtle changes cannot be precluded. Future studies of larger sample sizes are needed to detect possible statistical differences under smaller effect size. We had investigated the global network metrics and modular structure across a wide range of cost levels, using liberal, uncorrected statistical threshold for 38 cost levels to better detect the differences. Thus, it can be reasonable to conclude that there would be no significant differences in the graph metrics between the PDM and control groups.

Several points for further consideration should be addressed in our study. Firstly, the different atlas parcellation schemes may influence the results of topological network characteristics^62–64^, and the parcellation scheme of the cerebral cortex for graph theoretical network analysis requires further optimization and standardization^27,65,66^. The AAL and Harvard-Oxford cortical and subcortical probabilistic atlases are the parcellation schemes that are commonly used in graph theoretical network analysis of fMRI studies^36,67^. Secondly, it should be noted that the functional connectivity network in this study was based on the 90 cerebral regions of the AAL atlas, excluding the cerebellar regions. Although our previous PDM study showed *trait-related* changes of gray matter volume in the right cerebellar tonsil^6^, the specific role of the cerebellum in pain processing and modulation is not completely understood^68^. Notwithstanding, technical challenges for image preprocessing of the cerebellum (e.g., normalization procedure to the atlas) arise due to the small size and functional heterogeneity of the cerebellum^69^. Finally, the young PDM females in our study group experienced moderate to severe menstrual pain with a wide spectrum of individual pain differences. Owing to the sample size, we did not perform subgroup analyses according to the pain severity (i.e., moderate and severe PDM). Future studies of larger sample sizes to address the relationship between the topological profiles and the clinical characteristics (e.g., pain severity) can be important to disclose the intricacy between imaging endophenotypes and clinical phenotypes of PDM.

## Conclusions

Our current study indicates that, despite regional dysfunction and structural alterations, the overall integrity of intrinsic functional brain architecture in young PDM females, in terms of global and regional network metrics and modular structure, showed no differences compared to females without PDM at our detection thresholds (Cohen’s *d*_*between-group*_ ≥ 0.52 and Cohen’s *d*_*within-group*_ ≥ 0.37). Although we cannot completely preclude small to medium effect size differences of functional brain architecture owing to the underpowered concern, it is plausible that the absence of significant changes in the functional brain architecture allows young PDM females to maintain normal psychosocial outcomes during the pain-free follicular phase.

## Methods

### Subjects

The subjects of this study (59 PDM and 68 control females) were a subset of the participants (smaller in the sample size) from our previous genetic association and behavioral study of PDM^8^ who had completed the whole study protocols and were eligible for neuroimaging analyses in this study. Two PDM and 6 control females were excluded for further analyses owing to the significant head motion (translation >2 mm or rotation >2°) during the MRI scan. Eventually, 57 otherwise healthy females with PDM (age, 23.1 ± 2.27 years) and 62 education-matched, healthy control females (age, 23.7 ± 2.40 years) were recruited for the present study (see Table 1 for demographic data). The inclusion criteria for subjects with PDM were as follows: 1) 20–30-year-old Taiwanese (Asian) females; 2) a regular menstrual cycle of approximately 27–32 days; 3) a history of menstrual pain longer than 6 months; 4) average menstrual pain under regular treatment with a rating higher than 4 on a verbal numerical scale (VNS, 0 = not at all, 10 = the worst imaginable pain) in the last 6 months; and 5) right-handedness, as confirmed by the Edinburgh Handedness Inventory^70^. All PDM females were clinically examined and diagnosed in the gynecology clinic by the same gynecologist (H.T.C.). All subjects in the PDM group received pelvic ultrasonography to exclude secondary dysmenorrhea caused by organic pelvic diseases, such as endometriosis or adenomyosis. The inclusion criteria for the healthy control females were similar to those for the PDM group, except that the subjects of the control group had no pain whatsoever during menses (VNS = 0). The exclusion criteria for all of the subjects were as follows: 1) using oral contraceptives, hormonal supplements, Chinese herbal medicine, or any centrally acting medication (e.g., opioid, anti-epileptics) within 6 months prior to the study; 2) pathological pituitary gland disease; 3) organic pelvic disease; 4) any psychiatric or neurological disorders (e.g., premenstrual dysphoric disorder); 5) any head injury with loss of consciousness or brain surgery; 6) immediate plans for pregnancy or a positive pregnancy test; 7) a history of childbirth; and 8) having a metal/pacemaker implant, claustrophobia, or any contraindications in relation to MRI. No analgesics had been taken by the subjects within 24 hours before the study. Some of these subjects have been analyzed in the previously published neuroimaging studies^9,11,12^. The overall study was conducted in accordance with the Declaration of Helsinki and was approved by the Institutional Review Board of Taipei Veterans General Hospital. All subjects provided written informed consent.

### Experimental design

MRI scanning was individually scheduled according to the first day of menstruation for each subject. All of the subjects received psychological assessments, blood sampling for gonadal hormone assays, and brain MRI scanning (T1 and resting-state fMRI images) during the MENS phase (days 1–3 of the menstrual cycle) and POV phase (days 12–16 of the menstrual cycle). Ovulation was confirmed using a urinary luteinizing hormone test (Han Chiun Proper LH Rapid Test).

### Psychological assessments

To evaluate the psychological status throughout the menstrual cycle, all of the subjects in the two groups completed self-reported psychological measurements, including the Spielberger State-Trait Anxiety Inventory^71^, the Beck Anxiety Inventory^72^, the Beck Depression Inventory^73^, and the Pain Catastrophizing Scale^74^, during the respective MENS and POV phases. The McGill Pain Questionnaire was completed by the PDM females at the inception stage and the MENS phase during the experiment to assess their respective overall and current experiences of menstrual pain.

### Serum gonadal hormone measurements

The sera extracted from the blood samples drawn during the respective MENS and POV phases were stored for batch analysis using commercialized assays (UniCel DxC 800 Synchron Clinical Systems, Beckman Coulter, Inc., Brea, CA). The total serum concentrations were assayed using a chemiluminescence immunoassay technique for estradiol and progesterone and a radioimmunoassay technique for testosterone.

Our previous report revealed: 1) there were significant main effects of group and menstrual cycle phase as well as the interaction between them on the psychological measurements and 2) there was a significant main effect of menstrual cycle phase, but no main effect of group or interaction between group and menstrual cycle phase, on the serum gonadal hormone levels^8^. For the purpose of this neuroimaging subset study, we only conducted the two-sample *t*-test to examine the between-group differences of the psychological and gonadal hormone measurements in each phase using SPSS Statistics 20.0 (SPSS Inc., Chicago, IL), without testing the overall interaction effects between group and menstrual cycle phase. This aim was to ascertain that the imaging data was not confounded by hormonal differences between the groups. The results were considered significant when *P* < 0.05. For more comprehensive statistical analyses and results of psychological and gonadal hormone measurements, please refer to our previous report^8^.

### Image acquisition

Resting-state fMRI images were acquired on a 3.0 Tesla MRI scanner (Magnetom Trio Tim, Siemens, Erlangen, Germany), using echo-planar imaging (EPI), with the following scanning parameters: repetition time (TR) = 2500 ms, echo time (TE) = 30 ms, 40 axial slices/image volume with slice thickness = 3.4 mm, flip angle = 90°, field of view (FOV) = 220 × 220 mm^2^, matrix size = 64 × 64, and voxel size = 3.4 × 3.4 × 3.4 mm^3^. The duration of the EPI scan was 507 sec, and it consisted of 200 volumes. All subjects were scanned with their eyes open in a supine and relaxed position. T1-weighted 3-dimensional structural images for each subject were acquired using a magnetization-prepared rapid-acquired gradient echo sequence (MPRAGE) with the following scanning parameters: TR = 2530 ms, TE = 3.03 ms, inversion time (TI) = 1100 ms, flip angle = 7°, FOV = 224 × 256 mm^2^, matrix size = 224 × 256, and slice thickness = 1 mm. To reduce interference from head motion and to reduce ambient noise levels, head cushions and earplugs were used, respectively.

### Image preprocessing

All EPI images were preprocessed using Statistical Parametric Mapping software (SPM8, Wellcome Trust Centre for Neuroimaging, University College London, London, United Kingdom, http://www.fil.ion.ucl.ac.uk/spm) in MATLAB (The MathWorks, Inc., Natick, MA, USA) with the following procedures: the correction of slice timing, the realignment for head motion correction (6-parameter rigid body transformation), and the spatial normalization. The time course of head motion for each subject was obtained by estimating the translation and rotation in each axis for the 200 consecutive EPI volumes. Because head motion has a significant influence on intrinsic functional connectivity measurements^75^, we excluded subjects with significant head motion (translation >2 mm or rotation >2°) of any volume from further analysis. The EPI images were spatially normalized using the SPM’s standard EPI template in Montreal Neurological Institute (MNI) space and re-sampled to an isotropic 2 × 2 × 2 mm^3^ voxel size. For the time series of blood oxygen level-dependent (BOLD) signals in each voxel of the normalized image, the effects of head motion (the 6 motion parameters estimated from rigid-body realignment) and the signals of white matter regions and ventricular system were removed by linear regression, and the shift of BOLD signals was detrended. The band-pass filter was set at 0.01–0.08 Hz for the resulting time series to extract the low-frequency oscillating components that contributed to resting-state functional connectivity^76^. Global signal regression was not performed due to the concern of potential distortion on correlation patterns^77,78^.

### Construction of connectivity network

Brain functional networks, composed of nodes (parcellated brain regions) and edges (inter-nodal functional connectivity), were constructed from resting-state fMRIs.

#### Definition of node

The node of the network was defined as the parcellated brain regions according to the AAL atlas (consisting of 90 cerebral and 26 cerebellar anatomical regions)^31^. For the nomenclature of the 90 parcellated cerebral regions (45 areas per hemisphere) used in network construction, please refer to our previous report^79^. The resulting templates of the 90 nodes were then respectively co-registered with the preprocessed fMRI images of each subject. The mean time series of each node was calculated by averaging the time series of each voxel in that node.

#### Definition of edge

The edge of the network was defined as the degree of correlation (Pearson correlation coefficient, *r*) between the mean time series of each pair of nodes^22,24,26,27,79^. To obtain a better normality of the correlation, the 90-by-90 ‘*r*-value matrix’ was converted to a ‘z-score matrix’ using Fisher’s r-to-z transformation. The weight of the edge was defined as the absolute value of the z-score matrix.

The z-score matrix was then thresholded at different levels of sparsity using the minimum spanning tree method, followed by global thresholding^80^. The minimum spanning tree method aims to force the connectedness of sparse or low-density graphs and to prevent the emergence of fragmented networks with isolated islands, which are neuroscientifically unexplainable and unjustified. For each node, the edge of the highest weight was retained to keep the node connected with at least one neighboring node (i.e., local thresholding). The step of local thresholding was iterated until all nodes were able to connect with one another, giving rise to a fully connected backbone network without the emergence of isolated islands. The edges of the strongest weight, ranked by all the edges, were sequentially added into the backbone network. This ‘growing’ method was iterated until the number of edges meets with the assigned network sparsity (i.e., global thresholding). To investigate the effect of different network sparsity on graph metrics, weighted and binary networks were constructed with cost values ranging from 0.03 to 0.40, at increments of 0.01. For a weighted network, the edge weight exceeding the threshold was retained, otherwise it was discarded. For a binary network, the edge weight exceeding the threshold was set to ‘1’, otherwise it was set to ‘0’. Binary networks contain only the information whether an edge (connection) between two nodes is present or not according to the preset threshold. Weighted networks contain not only the information whether an edge is present or not, but also the connection strength between two nodes. Binary networks are succinct to characterize and compute the organizational characteristics^29^. Weighted networks can, compared to the binary networks, retain more biologically relevant information and provide different but complementary information of network organization^29,81^. We used both binary and weighted network constructions to cross-validate the results.

### Network analysis software

The global and regional network metrics and modularity of brain functional networks were computed using the Brain Connectivity Toolbox^29^ (https://sites.google.com/site/bctnet/) in MATLAB. Between-group differences of network community structures (i.e., similarity analysis), in terms of modular partitions and modular assignment of specific nodes, were examined according to the approach reported by Alexander-Bloch *et al*.^82^. We adopted the R codes provided by the authors and computed the NMI and Pearson’s phi for the similarity analyses of modular partitions and modular assignment of specific nodes, respectively. For the codes to perform the similarity analyses please refer to the Supplementary information. The similarity comparisons were carried out by means of permutation procedure for the planned contrast. We compared the average within-group similarity of the actual data to that of the permuted data where the group memberships are randomized. The *P* value is defined as the number of instances that the permuted within-group similarity exceeds the actual within-group similarity, divided by the number of permutations. Significant group differences are indicated should the within-group similarity of the actual data always exceed that of the permuted data^82^. For the details of computational processes, please refer to the respective sections (Modular structure & Modular assignment of specific nodes).

### Global network metrics of the resting-state functional network

The mean clustering coefficient^83,84^, characteristic path length^83^, global efficiency^85^, and local efficiency^85^ of the network were assessed for each of the MENS and POV phases in the PDM and control groups. The mean clustering coefficient of the network represents the tendency of network nodes to form local clusters (i.e., local connectedness), and the characteristic path length indicates how well the network nodes are interconnected (global connectedness)^34^. Small-worldness reflects the combination of a high clustering coefficient and a short characteristic path length^83^. A high clustering coefficient characterizes an efficient local information processing (high local efficiency) and a short characteristic path length features a high level of global communication efficiency (high global efficiency)^28,86^.

### Modular structure

The modularity^87,88^, the number of partitioned modules, as well as the similarity between two modular partitions were assessed. The modular structure is defined as a subset of nodes that are more densely interconnected among them than with other nodes within the network, indicating the formation of sub-networks^24^. Modularity is a measure of degree to which a network can be subdivided into smaller sub-networks^26^.

The global network metrics (mean clustering coefficient, characteristic path length, global efficiency, and local efficiency) and modular structure (modularity, number of partitioned modules, and similarity of modular partitions) of brain functional networks were assessed on the respective weighted and binary networks across a range of network cost (0.03–0.40). The statistical analyses for the mean clustering coefficient, characteristic path length, global efficiency, local efficiency, modularity, and number of partitioned modules of the network at a specific cost level were conducted using SPSS Statistics 20.0. Linear mixed models were used to examine the fixed effects of group (PDM vs. control) and menstrual cycle phase (MENS phase vs. POV phase), as well as the interaction between them. Subject was entered as a random effect. As menstrual cycle phase (i.e., gonadal hormones) may have an influence on the resting-state functional connectivity between brain regions^89^ and PDM females exhibit minimal or mild depressive and anxious symptoms during the MENS phase (see Results), the hormonal fluctuations (estradiol, progesterone, and testosterone levels) and psychological assessments (the Spielberger State-Trait Anxiety Inventory, the Beck Anxiety Inventory, and the Beck Depression Inventory scores) were entered as covariates in the statistical model. The variance–covariance matrix was assumed to be unstructured since there were no presumptions on the correlation pattern of graph metrics between menstrual cycle phases. Significance was initially set at a threshold of corrected *P* < 0.05 (FDR correction for multiple comparisons of 38 cost levels), and no significant differences were found. Hence, we further lowered the significance level at a liberal threshold of uncorrected *P* < 0.05 (uncorrected for multiple comparisons of 38 cost levels) to better the detection of possible differences.

Similarity between two modular partitions (quantified by the NMI^82,90^) was compared by a permutation procedure^82^. The NMI, a similarity index, can be used to evaluate different modular partition algorithms and to estimate the similarity of modular structures across subjects^90^. The NMI value ranges from 0 to 1, where 1 denotes that the two modular partitions are identical and 0 that they are totally independent^82^. For the equation of NMI, please refer to previous reports^82,90^. Between-group comparisons for each phase and between-phase comparisons for each group were computed to address the *state* and *trait* effects of dysmenorrhea on the similarity of modular structure of brain functional networks^11,12^. The alterations of modular partitions during the MENS phase in the between-group comparison (MENS phase of PDM vs. MENS phase of control) and in the between-phase comparison for the PDM group (MENS phase of PDM vs. POV phase of PDM) were regarded as *state-related* changes. The alterations during the POV phase in the between-group comparison (POV phase of PDM vs. POV phase of control) were regarded as *trait-related* changes. At a specific cost level, the average pairwise NMIs during the same menstrual cycle phase were separately calculated across subjects within the control and PDM groups. We tested the hypothesis that the average within-group pairwise similarity is higher than the average between-group pairwise similarity, which exhibits a genuine difference in similarity of modular partitions between the control and PDM groups during the same menstrual cycle phase^82,91^. The permutation procedure to compare the similarity of modular partitions was performed by varying the group membership, with 10000 permutations^82^. Significance was set at a stringent threshold of corrected *P* < 0.05 (FDR correction for multiple comparisons of 38 cost levels) initially, and no significant differences were found. Hence, we further lowered the significance level at a liberal threshold of uncorrected *P* < 0.05 (uncorrected for multiple comparisons of 38 cost levels) to unravel possible subtle differences.

### Modular assignment of specific nodes

For the analyses of modular assignment of specific nodes, we analyzed the respective weighted and binary networks with cost values ranging from 0.03 to 0.40. To investigate the difference in modular assignment of a specific node of interest (NOI) at a specific cost level during the same menstrual cycle phase, the similarity of module labels of two subjects, in terms of the network community of the NOI, was calculated. For a given NOI, for each subject, all of the other nodes were labeled ‘1’ if they shared the same module with the NOI, and ‘0’ if not. The similarity of the module labels between each pair of subjects was calculated using Pearson’s phi, a method of Pearson correlation for dichotomous variables. For the given NOI, a higher phi value indicates a higher similarity of modular assignment between subjects. We tested the hypothesis that the average within-group pairwise similarity is higher than the average between-group pairwise similarity, which exhibits a genuine difference in similarity of modular assignment of specific nodes between the control and PDM groups during the same menstrual cycle phase^82^. The permutation procedure to compare the similarity was performed by varying the group membership, with 10000 permutations^82^. The permutation tests were performed for every one of the 90 AAL nodes at each of the 38 cost levels. Under the premise of equivalent statistical stringency for the global network metrics and modular assignment of specific nodes, FDR correction was only applied to correct for multiple comparisons of 90 AAL nodes, but not for multiple comparisons of 38 cost levels.

### Regional network metrics of the resting-state functional network

For each node, the regional network metrics (local efficiency, clustering coefficient, and degree) were assessed on the respective weighted and binary networks across a range of network cost (0.03–0.10, at increments of 0.01) for each of the MENS and POV phases in the PDM and control groups. Low cost levels were selected for network construction to avoid spurious or statistically non-significant connections between nodes^28,29^. Node degree is defined as the number of links connected to a node^29^. The node with a high degree is interacting with many other nodes in the brain functional network^29^.

Data were analyzed using SPSS Statistics 20.0. At a specific cost level, linear mixed models were used to examine the fixed effects of group (PDM vs. control) and menstrual cycle phase (MENS phase vs. POV phase), as well as the interaction between them on the regional network metrics of each node. The hormonal fluctuations (estradiol, progesterone, and testosterone levels) and psychological assessments (the Spielberger State-Trait Anxiety Inventory, the Beck Anxiety Inventory, and the Beck Depression Inventory scores) were entered as covariates in the statistical model. The variance–covariance matrix was assumed to be unstructured. FDR correction was applied to correct for multiple comparisons of 90 AAL nodes, but not for multiple comparisons of 8 cost levels.

### Robustness of methodological variation

To validate our observations using results obtained from different methodological approaches, we analyzed the difference in the global and regional network metrics and modular structure using the networks constructed by parcellated brain regions according to the Harvard-Oxford cortical and subcortical probabilistic atlases. The atlases are composed of 48 cortical and 7 subcortical brain regions in each hemisphere, and a total of 110 nodes (55 areas per hemisphere) were used in the network construction.

## Electronic supplementary material

Supplementary Information

Table S1

Table S2

Table S3

Table S4

NMI_calculation

NMI_permutation
