# Supplementary material for: Unaltered intrinsic functional brain architecture in young women with primary dysmenorrhea
Source: Sci Rep. 2018 Aug 28;8:12971. doi: 10.1038/s41598-018-30827-6 (PMC6113269; doi:10.1038/s41598-018-30827-6)

Unaltered intrinsic functional brain architecture in young women with primary dysmenorrhea

Lin-Chien Lee[1,2,3,#], Yueh-Hua Chen[1,2,#], Chia-Shu Lin[4], Wei-Chi Li[1], Intan Low[5], Cheng-Hao Tu[1,2,6], Chih-Che Chou[2], Chou-Ming Cheng[2], Tzu-Chen Yeh[1,2,7], Li-Fen Chen[1,2,5], Hsiang-Tai Chao[8,9] & Jen-Chuen Hsieh[1,2,*]

[1]Institute of Brain Science, School of Medicine, National Yang-Ming University, Taipei, Taiwan

[2]Integrated Brain Research Unit, Division of Clinical Research, Department of Medical Research, Taipei Veterans General Hospital, Taipei, Taiwan

[3]Department of Physical Medicine and Rehabilitation, Cheng Hsin General Hospital, Taipei, Taiwan

[4]Department of Dentistry, School of Dentistry, National Yang-Ming University, Taipei, Taiwan

[5]Institute of Biomedical Informatics, School of Medicine, National Yang-Ming University, Taipei, Taiwan

[6]Graduate Institute of Acupuncture Science, College of Chinese Medicine, China Medical University, Taichung, Taiwan

[7]Department of Radiology, Taipei Veterans General Hospital, Taipei, Taiwan

[8]Department of Obstetrics and Gynecology, School of Medicine, National Yang-Ming University, Taipei, Taiwan

[9]Department of Obstetrics and Gynecology, Taipei Veterans General Hospital, Taipei, Taiwan

I. Supplementary results: Analysis of head motion parameters.

We conducted repeated-measures ANOVAs on the root mean squares of both overall translation and rotation parameters estimated using the formula in the published study[1]. No main effect of group (primary dysmenorrhea vs. control), menstrual cycle phase (menstrual phase vs. periovulatory phase), or interaction between them was noted for the overall translation and rotation parameters of head motion (all $P > 0.05$).

Reference:

1. Liu, Y. *et al.* Disrupted small-world networks in schizophrenia. *Brain.* **131**, 945-961 (2008).

II. Supplementary results: Regional network metrics of the resting-state functional network.

For the clustering coefficient, node degree, and local efficiency of each node of the respective weighted and binary networks constructed by parcellated brain regions according to the Automated Anatomical Labeling (AAL) and Harvard-Oxford cortical and subcortical probabilistic atlases, see **Supplementary Table S1**, **S2**, **S3**, and **S4** online.

III. Supplementary results: The global network metrics and modular structure on the respective weighted and binary networks constructed by parcellated brain regions according to the Harvard-Oxford cortical and subcortical probabilistic atlases.

The findings derived from the Harvard-Oxford cortical and subcortical probabilistic atlases were consistent with those derived from the AAL atlas [**Fig. S1**, **S2**, **S3**, **S4**, **S5**, and **S6**].

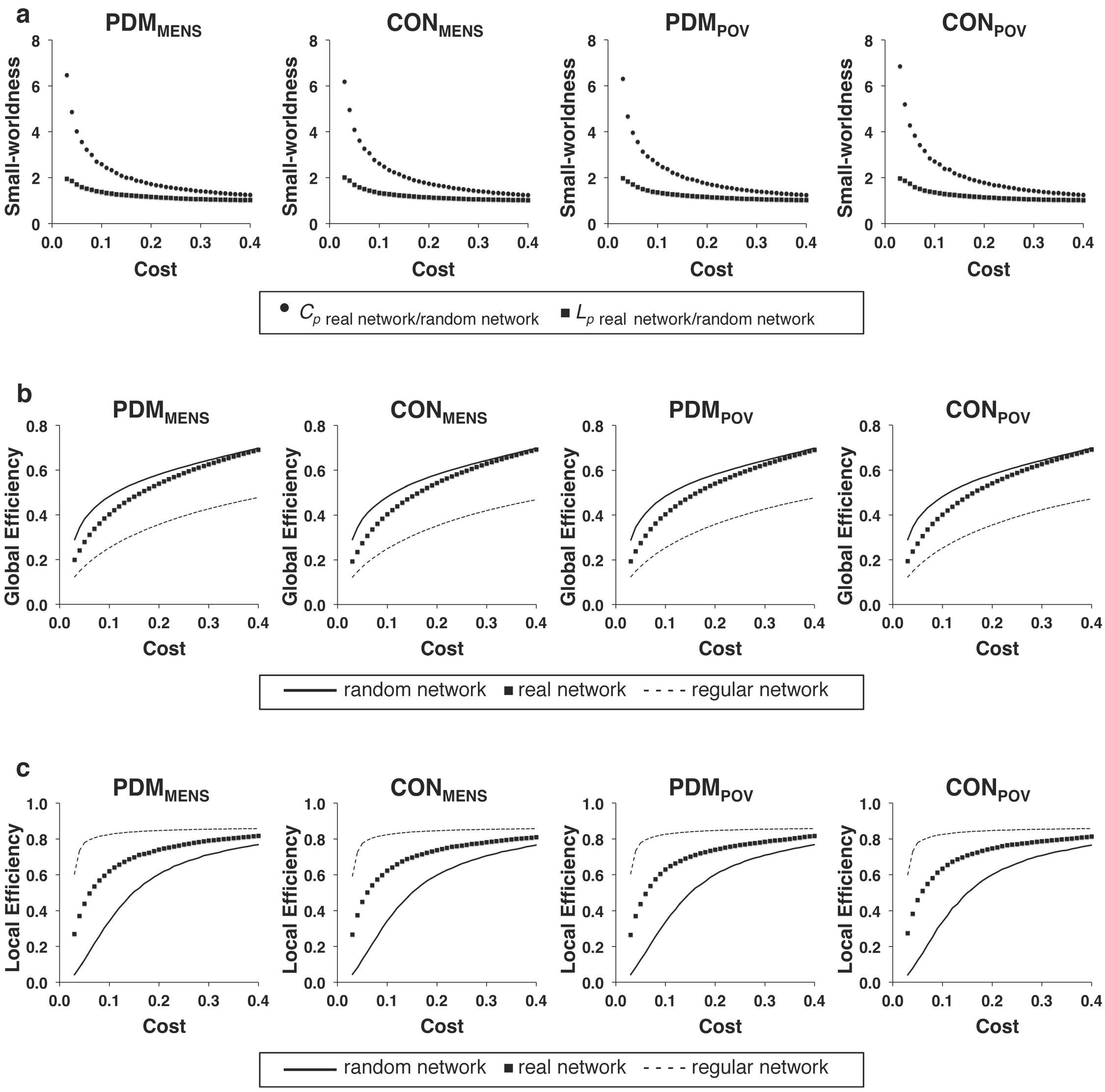

Figure S1. The global network metrics of the weighted network.

The global network metrics of (**a**) small-worldness (C_p and L_p), (**b**) global efficiency, and (**c**) local efficiency in the weighted network are plotted over the range of network costs (0.03-0.40). No significant differences were found in the global network metrics among the PDM_{MENS}, CON_{MENS}, PDM_{POV}, and CON_{POV} by conducting linear mixed models. C_p, clustering coefficient; L_p, characteristic path length; CON_{MENS}, menstrual phase of the control group; CON_{POV}, periovulatory phase of the control group; PDM_{MENS}, menstrual phase of the primary dysmenorrhea group; PDM_{POV}, periovulatory phase of the primary dysmenorrhea group.

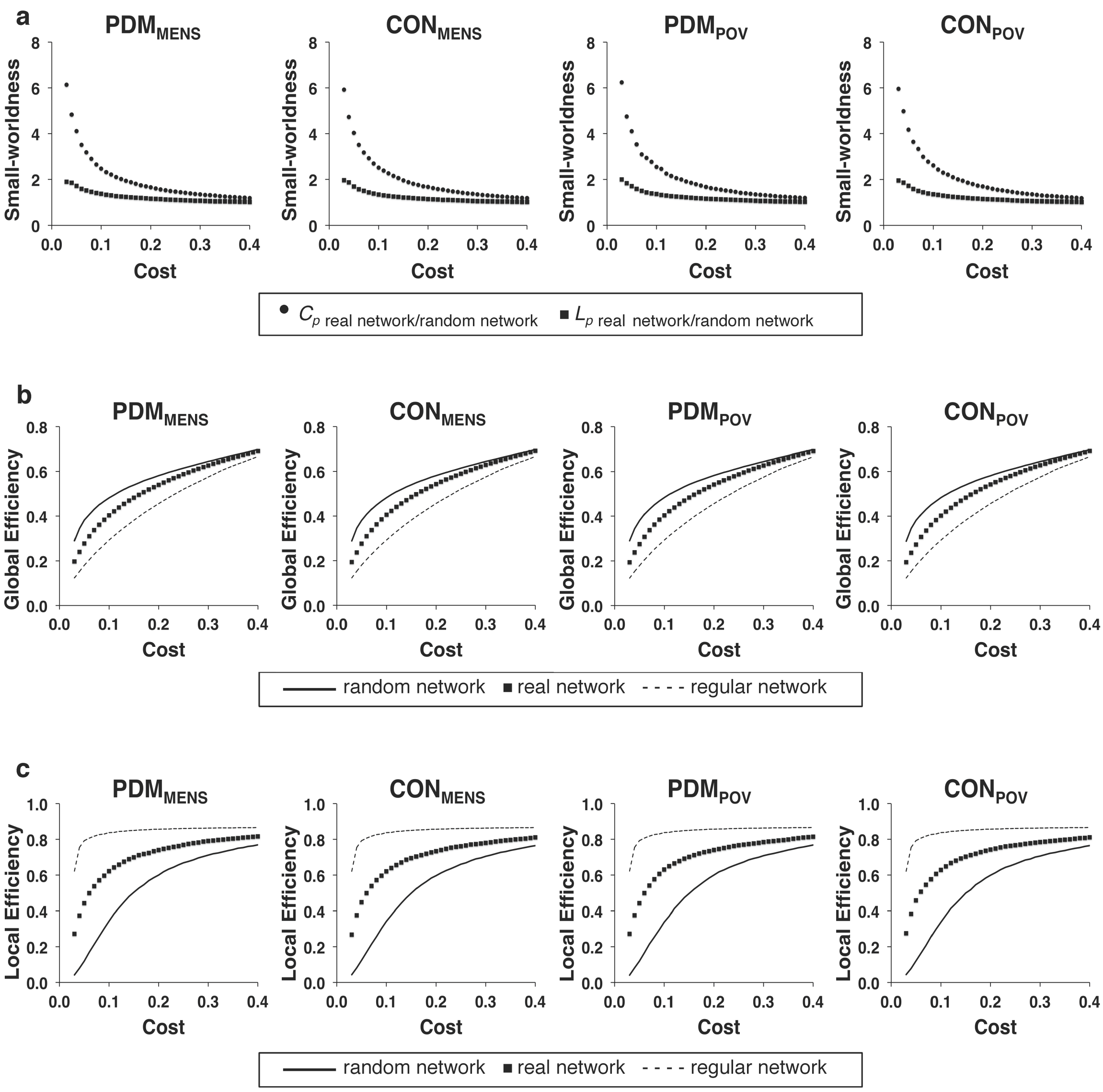

Figure S2. The global network metrics of the binary network.

For the full range of network costs (0.03-0.40) of the binary network, no significant differences were found in the global network metrics among the PDM_{MENS}, CON_{MENS}, PDM_{POV}, and CON_{POV} by conducting linear mixed models. C_p, clustering coefficient; L_p, characteristic path length; CON_{MENS}, menstrual phase of the control group; CON_{POV}, periovulatory phase of the control group; PDM_{MENS}, menstrual phase of the primary dysmenorrhea group; PDM_{POV}, periovulatory phase of the primary dysmenorrhea group.

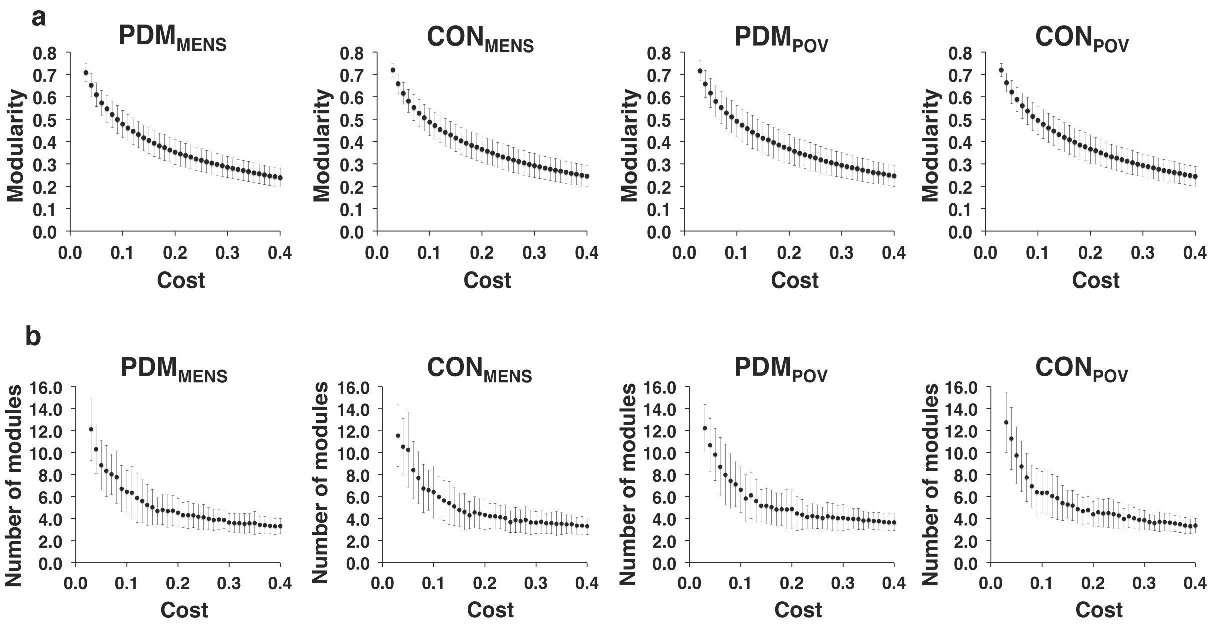

Figure S3. The modular structure of the weighted network.

For the full range of network costs (0.03-0.40) of the weighted network, no significant differences were found in (**a**) the modularity and (**b**) the number of partitioned modules among the PDM_{MENS}, CON_{MENS}, PDM_{POV}, and CON_{POV} by conducting linear mixed models. The bar denotes the standard deviation of means. CON_{MENS}, menstrual phase of the control group; CON_{POV}, periovulatory phase of the control group; PDM_{MENS}, menstrual phase of the primary dysmenorrhea group; PDM_{POV}, periovulatory phase of the primary dysmenorrhea group.

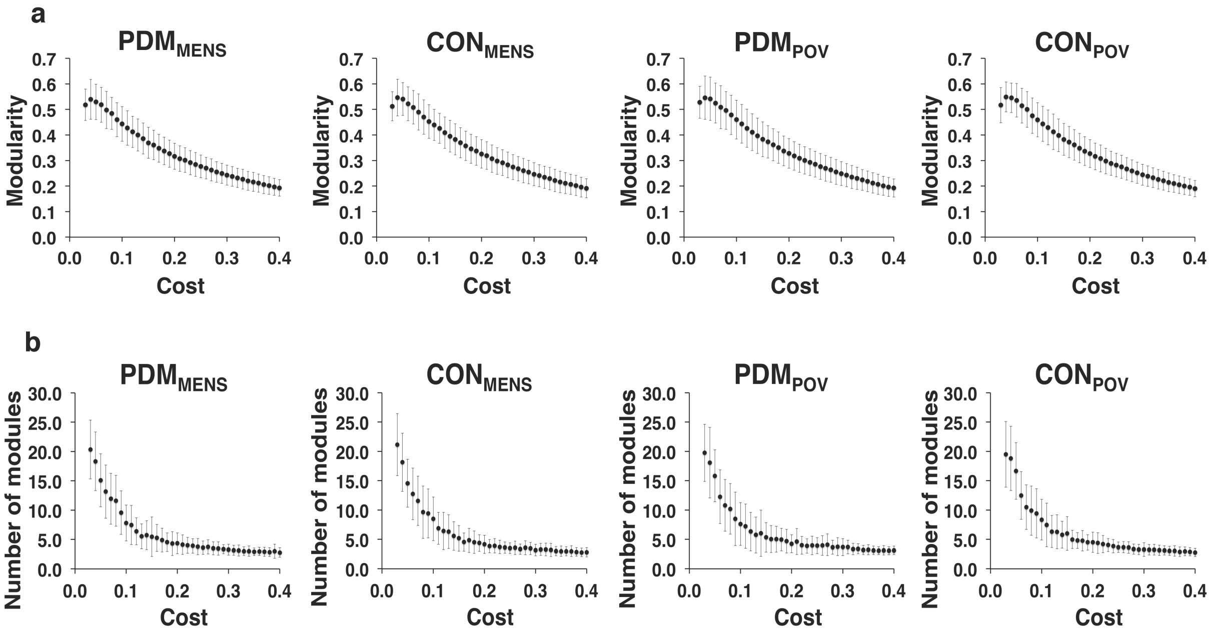

Figure S4. The modular structure of the binary network.

For the full range of network costs (0.03-0.40) of the binary network, no significant differences were found in (**a**) the modularity and (**b**) the number of partitioned modules among the PDM_{MENS}, CON_{MENS}, PDM_{POV}, and CON_{POV} by conducting linear mixed models. The bar denotes the standard deviation of means. CON_{MENS}, menstrual phase of the control group; CON_{POV}, periovulatory phase of the control group; PDM_{MENS}, menstrual phase of the primary dysmenorrhea group; PDM_{POV}, periovulatory phase of the primary dysmenorrhea group.

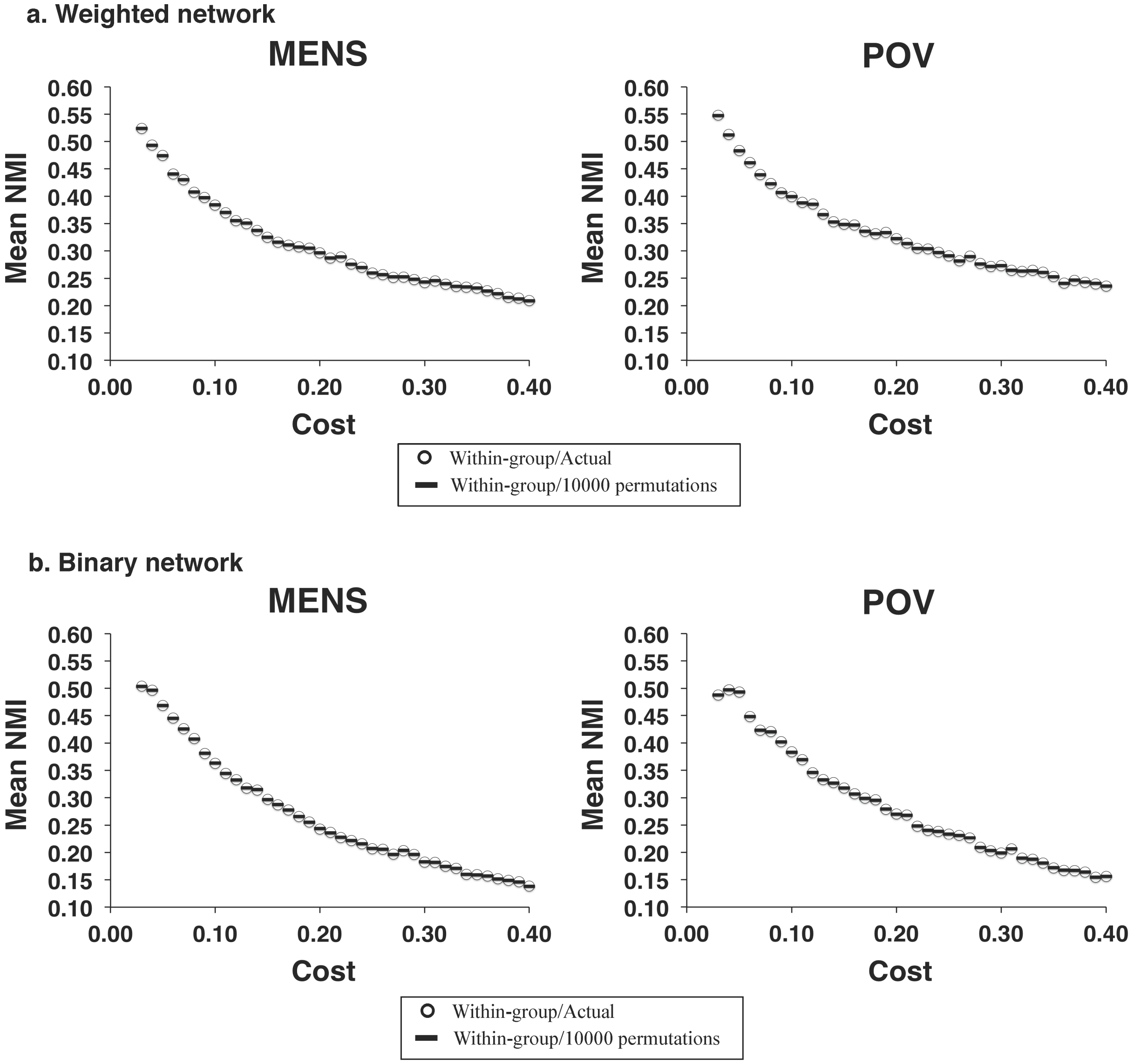

Figure S5. The similarity of modular partitions during the same menstrual cycle phases in the respective weighted and binary networks.

For the full range of network costs (0.03-0.40) of the respective (**a**) weighted and (**b**) binary networks, no significant differences were found in the similarity of modular structure for the between-group comparisons during each of the MENS and POV phases. MENS, menstrual phase; NMI, normalized mutual information; POV, periovulatory phase.

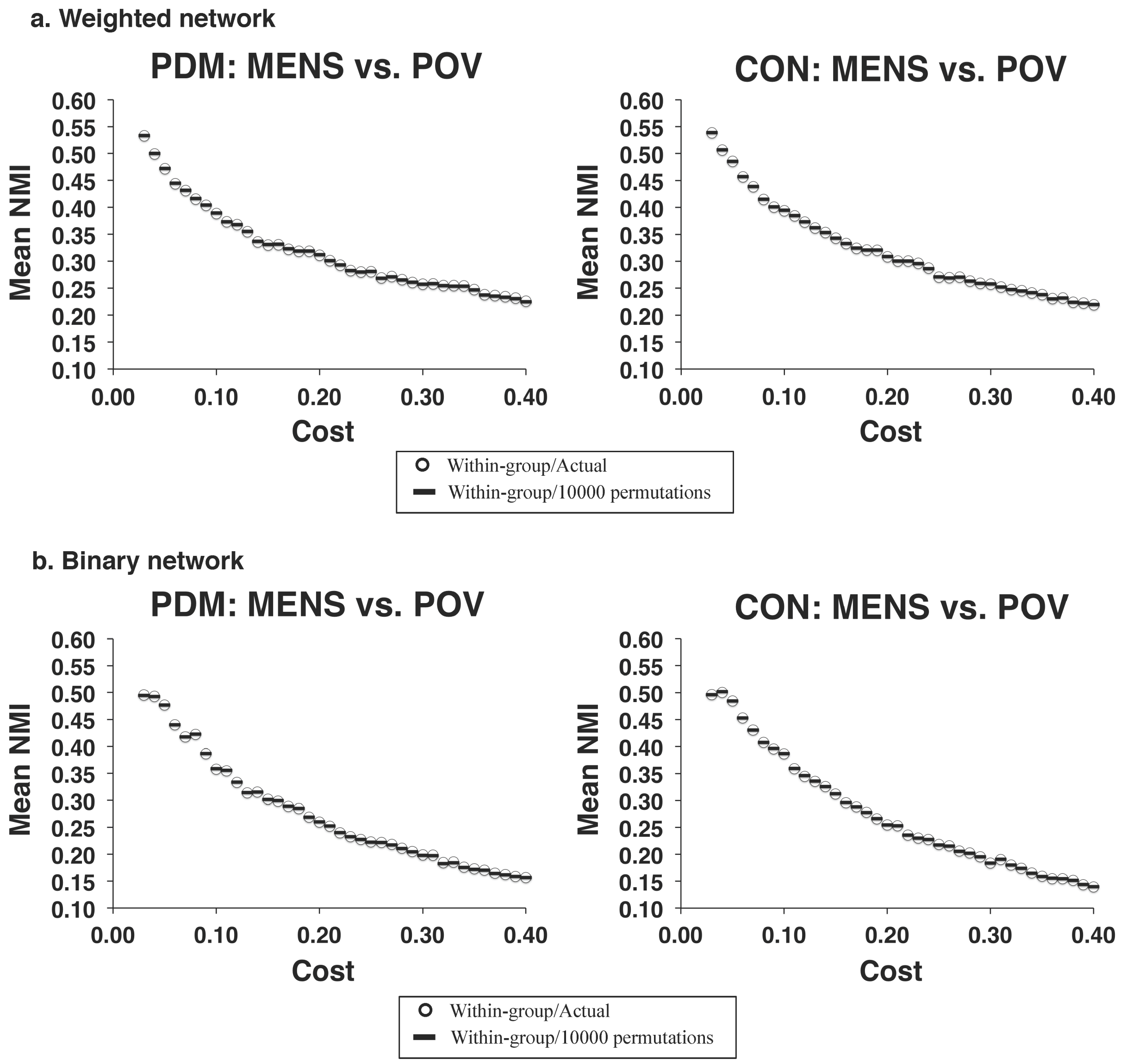

Figure S6. The similarity of modular partitions for the between-phase comparisons in the respective PDM and CON groups.

For the full range of network costs (0.03-0.40) of the respective (**a**) weighted and (**b**) binary networks, no significant differences were found in the similarity of modular structure for the between-phase comparisons in each of the PDM and CON groups. CON, control; MENS, menstrual phase; NMI, normalized mutual information; PDM, primary dysmenorrhea; POV, periovulatory phase.

IV. Supplementary results: Graph metrics as a function of correlation threshold on the functional connectivity between brain regions.

Weighted and binary networks of parcellated brain regions according to the AAL atlas were constructed with correlation threshold values ranging from 0.30 to 0.80, at increments of 0.05. For the respective weighted and binary networks, no main effect of group (primary dysmenorrhea vs. control), menstrual cycle phase (menstrual phase vs. periovulatory phase), or interaction between them was noted for the mean clustering coefficient, global efficiency, local efficiency, and modularity of the network by conducting linear mixed models (all $P > 0.05$, uncorrected for multiple comparisons of 11 correlation threshold levels) (**Fig. S7**: weighted network; **Fig. S8**: binary network). For the abbreviations used in the **Fig. S7** and **S8**, see the legend of **Fig. S1**.

Figure S7: weighted network

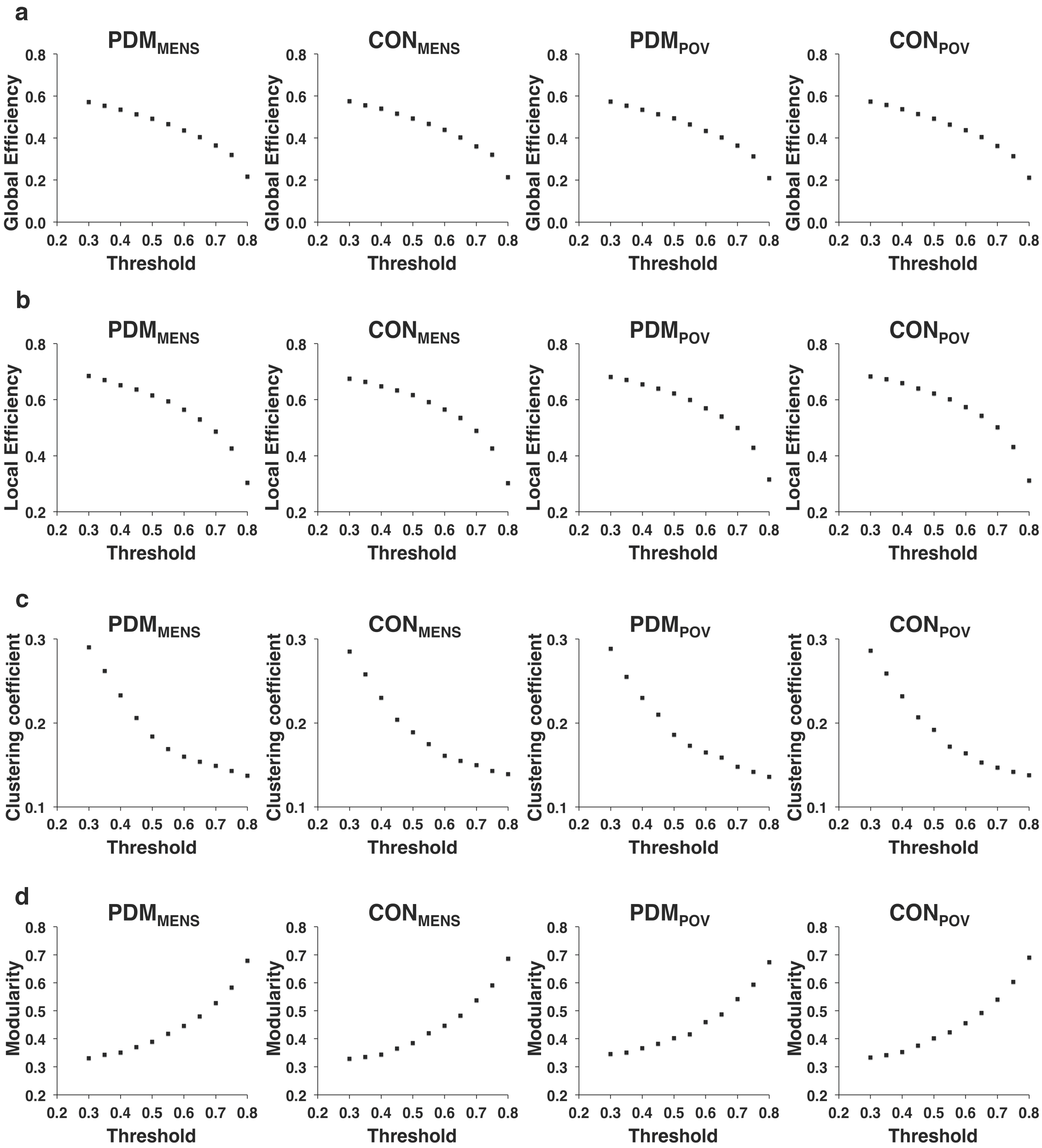

Figure S8: binary network

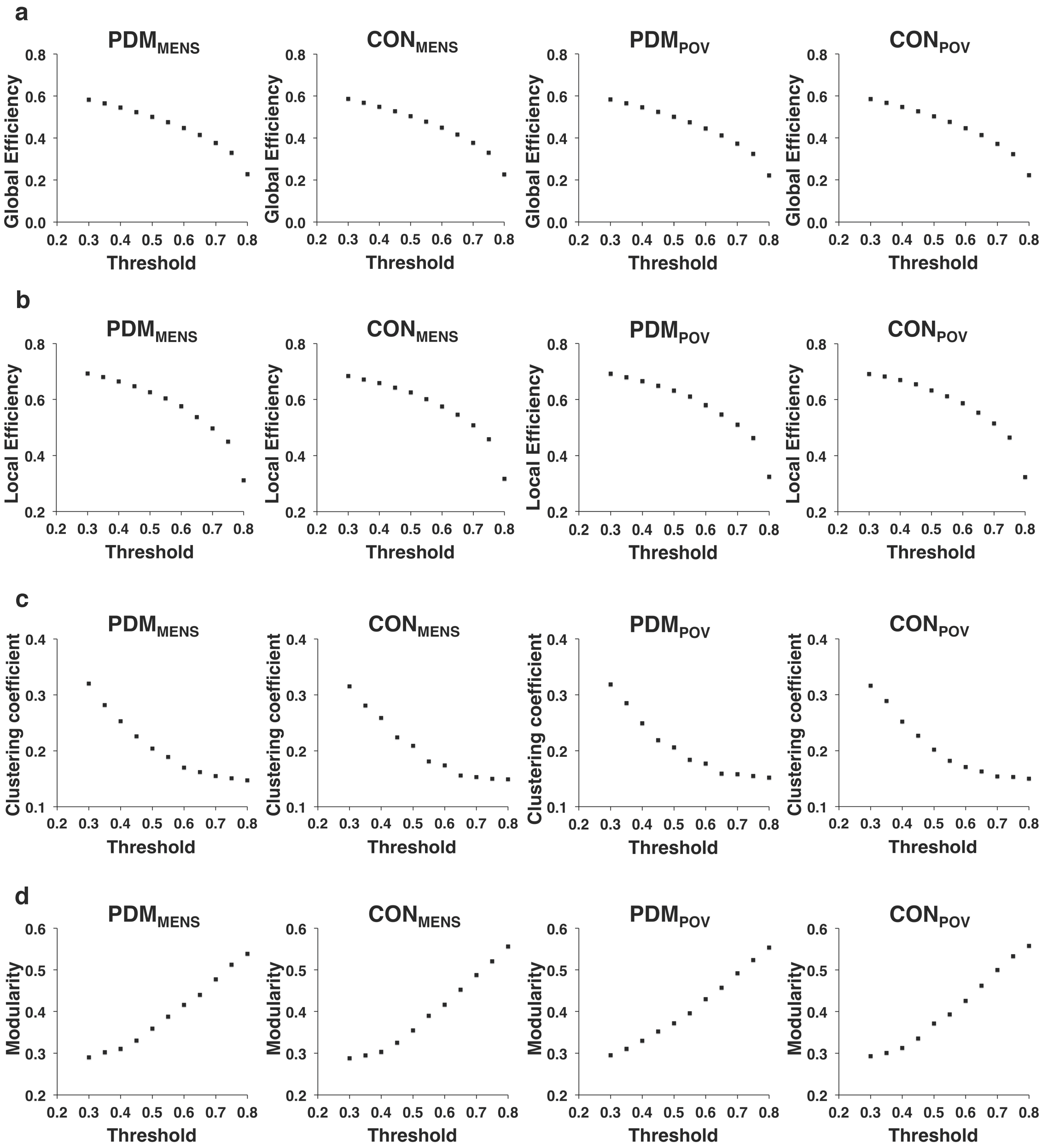

V. Supplementary results: Relationships between correlation threshold value (0.30 to 0.80, at increments of 0.05) and cost level (link density) in the respective weighted and binary networks of parcellated brain regions according to the AAL atlas (**Fig. S9**: weighted network; **Fig. S10**: binary network). For the abbreviations used in the **Fig. S9** and **S10**, see the legend of **Fig. S1**.

Figure S9: weighted network

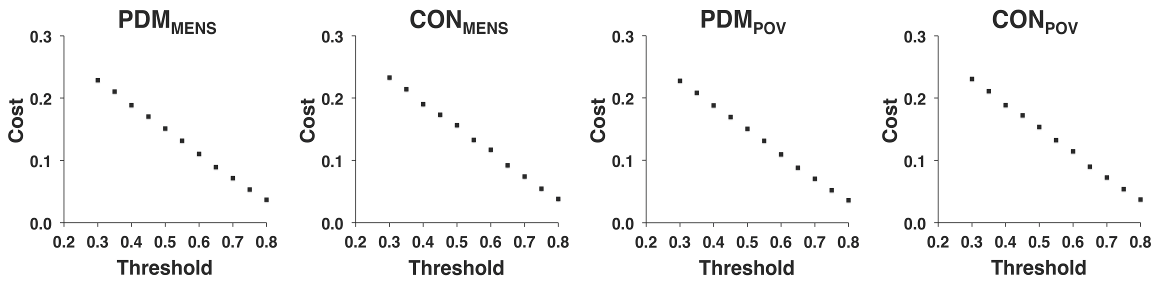

Figure S10: binary network

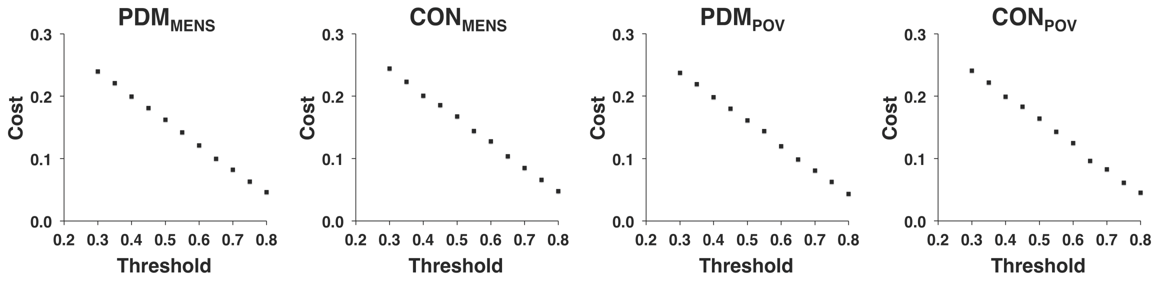

VI. Supplementary results: Relationships between cost level (link density) and the lowest correlation coefficient in the weighted network of parcellated brain regions according to the AAL atlas (**Fig. S11**). The highest correlation coefficient is 0.8939 for PDM_{MENS}, 0.9011 for CON_{MENS}, 0.8943 for PDM_{POV}, and 0.9087 for CON_{POV}. For the abbreviations used in the **Fig. S11**, see the legend of **Fig. S1**.

Figure S11

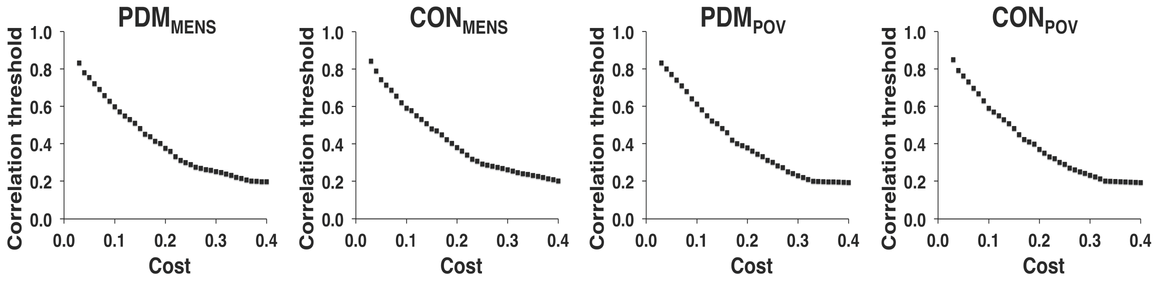

Supplement: Supplementary file 1 — Supplementary Information [file 41598_2018_30827_MOESM1_ESM.pdf]
